# The causal relationship between COVID-19 and estimated glomerular filtration rate: a bidirectional Mendelian randomization study

**DOI:** 10.1186/s12882-023-03443-4

**Published:** 2024-01-15

**Authors:** Qiuling Li, Mengjiao Lin, Yinghui Deng, Haozhang Huang

**Affiliations:** 1 Department of Nephrology, Blood Purification Center, Zhongshan City People’s Hospital, Zhongshan, 528403 China; 2 Department of Nephrology, Guangdong Provincial People’s Hospital, Guangdong Academy of Medical Sciences, Guangzhou, 510080 China; 3 Department of Cardiology, Guangdong Provincial People’s Hospital, Guangdong Cardiovascular Institute, Guangdong Academy of Medical Sciences, Guangzhou, 510080 China; 4 Department of Guangdong Provincial Key Laboratory of Coronary Heart Disease Prevention, Guangdong Provincial People’s Hospital, Guangdong Cardiovascular Institute, Guangdong Academy of Medical Sciences, Guangzhou, 510080 China; 5 The Second School of Clinical Medicine, Southern Medical University, Guangzhou, 510515 China

**Keywords:** Mendelian randomization, Coronavirus Disease 2019, Estimated glomerular filtration rate, Kidney function, Single nucleotide polymorphism

## Abstract

**Background:**

Previous Mendelian studies identified a causal relationship between renal function, as assessed by estimated glomerular filtration rate (eGFR), and severe infection with coronavirus disease 2019 (COVID-19). However, much is still unknown because of the limited number of associated single nucleotide polymorphisms (SNPs) of COVID-19 and the lack of cystatin C testing. Therefore, in the present study, we aimed to determine the genetic mechanisms responsible for the association between eGFR and COVID-19 in a European population.

**Methods:**

We performed bidirectional Mendelian randomization (MR) analysis on large-scale genome-wide association study (GWAS) data; log-eGFR was calculated from the serum levels of creatinine or cystatin C by applying the Chronic Kidney Disease Genetics (CKDGen) Meta-analysis Dataset combined with the UK Biobank (N = 1,004,040) and on COVID-19 phenotypes (122,616 COVID-19 cases and 2,475,240 controls) from COVID19-hg GWAS meta-analyses round 7. The inverse-variance weighted method was used as the main method for estimation.

**Results:**

Analyses showed that the genetically instrumented reduced log-eGFR, as calculated from the serum levels of creatinine, was associated with a significantly higher risk of severe COVID-19 (odds ratio [OR]: 2.73, 95% confidence interval [CI]: 1.38–5.41, *P* < 0.05) and significantly related to COVID-19 hospitalization (OR: 2.36, 95% CI: 1.39–4.00, *P* < 0.05) or infection (OR: 1.24, 95% CI: 1.01–1.53, *P* < 0.05). The significance of these associations remained when using log-eGFR based on the serum levels of cystatin C as genetically instrumented. However, genetically instrumented COVID-19, regardless of phenotype, was not related to log-eGFR, as calculated by either the serum levels of creatinine or cystatin C.

**Conclusions:**

Our findings suggest that genetical predisposition to reduced kidney function may represent a risk factor for COVID-19. However, a consistent and significant effect of COVID-19 on kidney function was not identified in this study.

**Supplementary Information:**

The online version contains supplementary material available at 10.1186/s12882-023-03443-4.

## Background

Coronavirus disease 2019 (COVID-19) imposed a significant burden on public health. As of the 20th of November 2022, there had been 634 million confirmed cases of COVID-19 globally, including 6.6 million deaths. Almost three years after the pandemic started, some patients infected with COVID-19 were found to suffer from long-term symptoms, a condition that is referred to as “long COVID.” This discovery poses a number of new clinical challenges [1–4].

Although COVID-19 is predominantly a respiratory disease, previous studies have reported a broad spectrum of kidney impairment during long COVID-19 [5, 6]. However, whether this association is causal remains unknown. Conventional observational studies may be influenced by a range of confounding factors, including socioeconomic status and drug use; consequently, such analyses may not provide definitive answers. Using genetic variation as a tool to predict exposure, Mendelian randomization (MR) provides an efficient tool that can determine estimates that are not influenced by confounding factors without any intervention [7–9]. Furthermore, investigating the distribution of single nucleotide polymorphisms (SNPs) by performing MR analysis can help to identify specific risk factors for the development of COVID-19 infection [10, 11]. This form of analysis may help us to identify groups of patients that may be more susceptible to renal dysfunction.

In a recent MR study [12], Zhao et al. reported that genetic predisposition to reduced estimated glomerular filtration rate (eGFR), as calculated from the serum levels of creatinine, was associated with increased susceptibility to severe COVID-19. Generally, the serum level of creatinine has become the most widely used parameter for evaluating kidney function in clinical practice [13]. However, since the serum levels of creatinine are influenced by muscle mass or diet, the calculation of eGFR from the serum levels of cystatin C presents certain advantages when assessing kidney function [14, 15]. Moreover, the additional trimming of SNPs is necessary to remove genetic variants that are likely to be related to creatinine metabolism instead of kidney function. Additionally, much is still unknown because of the limited number of associated single nucleotide polymorphisms (SNPs) of COVID-19 used in previous studies. It is necessary to determine the genetic association between eGFR and COVID-19 hospitalization or infection in a European population.

In the present study, we conducted an updated bidirectional MR analysis, including genome-wide summary statistics for log-eGFR, as calculated using the serum levels of creatinine or cystatin C, with a large sample size, the latest COVID-19 genome-wide association study (GWAS) data, and several sensitivity analyses, to investigate the assumptions generated by MR.

## Methods

### GWAS meta-analysis of COVID-19

**Fig. 1:**
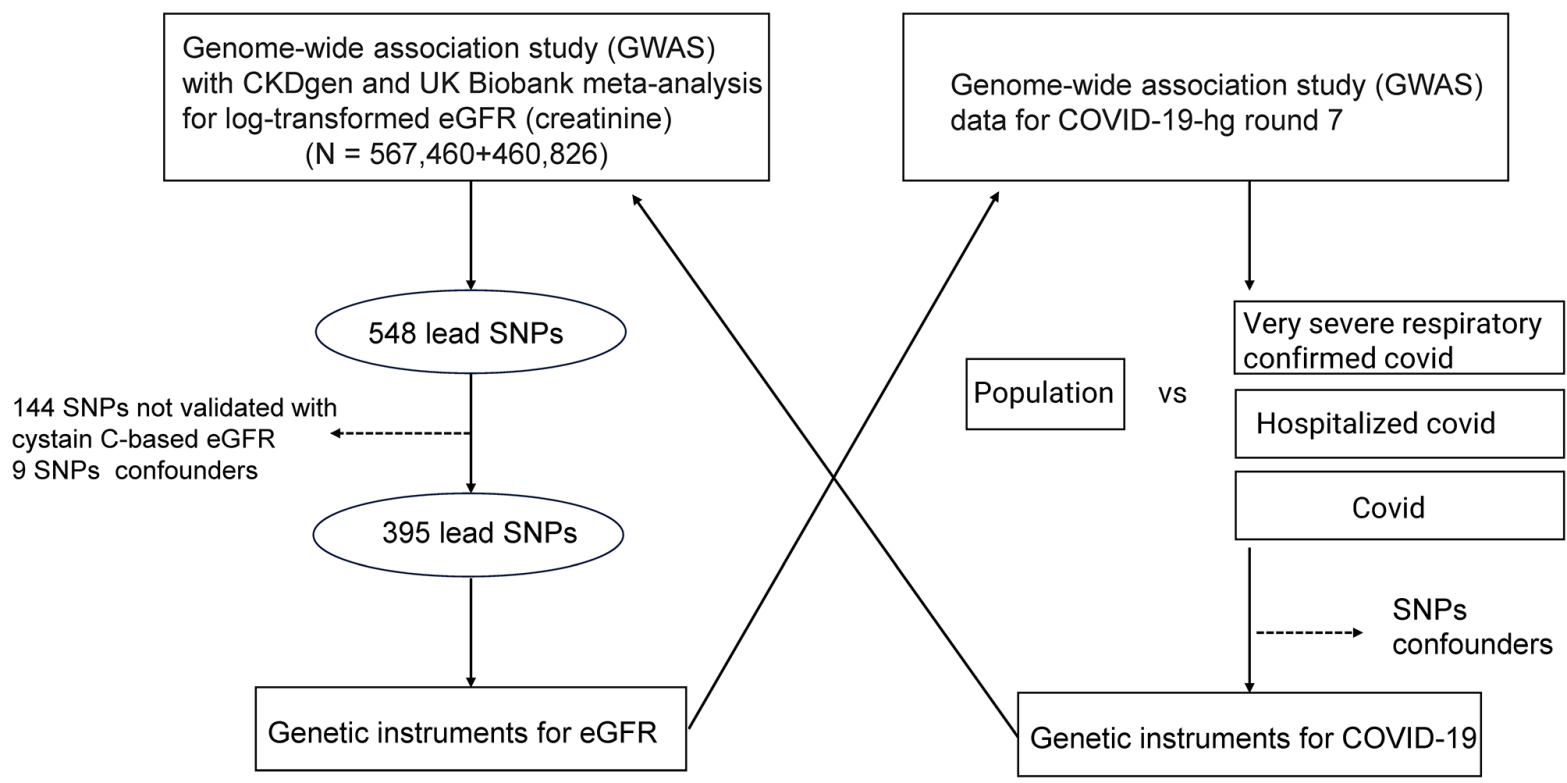
Overall design of the MR analysis in the present study

In the present study (Fig. 1), we used the latest release of COVID-19 GWAS data as exposure. SNPs in the genome-wide data that were significant (*P* < 5 × 10^–8^), uncorrelated (r^2^ < 0.01), and at least 1 Mbp apart, were considered as genetic variants from European samples in our bidirectional MR analysis. Confounding data were excluded due to the presence of associations (*P* < 5 × 10^–8^) with the risk of eGFR based on creatinine levels, including smoking status, arm fat and fat-free mass, and systolic and diastolic pressure (Supplementary Table 1).

Data relating to severe COVID-19 (13,769 cases and 1,072,442 controls) were derived from several large studies, including the UK Biobank, GENCOVID, GenOMICC, and BRACOVID. We also investigated COVID-19 hospitalization (32,519 cases and 2,062,805 controls) and all COVID-19 cases (122,616 cases and 2,475,240 controls) using the largest publicly available COVID-19 GWAS, mostly including individuals of European ancestry (details of the participating studies are given at https://www.covid19hg.org/results/r7/). Severe COVID-19 was defined as death or respiratory support following hospitalization due to COVID-19 as the primary reason for admission. COVID-19 hospitalization was defined as hospitalization due to coronavirus-related symptoms with laboratory-confirmed severe acute respiratory syndrome coronavirus 2 (SARS-CoV-2) infection. All COVID-19 cases were defined as (1) laboratory-confirmed SARS-CoV-2 infection (based on RNA and/or serology), (2) COVID-19 diagnosed by a physician, or (3) self-reported COVID-19. Participants in these cohorts who were not infected with COVID-19 were considered as controls. GWAS data were adjusted for age, age square, sex, the interaction between age and sex, and principal component [16].

For COVID-19 infection phenotype [17–20], we included 14,134 confirmed cases of COVID-19 with 1,284,876 controls. For hospitalized COVID-19 phenotype, we included 6,406 hospitalized COVID-19 cases with 902,088 controls as well as 1776 hospitalized cases with 6,443 non-hospitalized controls [23]. For severe COVID-19 phenotype, we included 4,792 confirmed cases of very severe respiratory infections with 1,054,664 population controls, 1,610 confirmed severe COVID-19 cases with respiratory failure and 2,180 population controls, and 182 confirmed critical illness cases with 910 population controls.

### GWAS meta-analysis of kidney function

A recent GWAS meta-analysis incorporating CKDGen and UK Biobank data with a genome-wide significant association featuring log-transformed eGFR data calculated from serum creatinine levels (eGFRcrea), and eGFR calculated from serum cystatin C levels (eGFRcys) in > 450,000 individuals was used to identify alternative biomarkers [21]. The increased sample size had an impact not only on GWAS findings for eGFR when calculated using serum creatinine levels (eGFRcrea) but also led to a substantial improvement in fine-mapping and alternative biomarker support.

A total of 548 SNPs were included in the MR analysis based on published genome-wide significant (*P* < 5 × 10^− 8^), and uncorrelated (r^2^ < 0.01) genetic variants, which were at least 1 Mbp apart. This analysis was used to predict eGFRcrea values for European samples when utilizing log-transformed eGFRcrea levels as an exposure. Additional trimming was required for these 548 SNPs. Thus, we removed the genetic variants of 144 SNPs that were likely to be related to creatinine metabolism instead of kidney function and showed different directions of regressed betas. We also removed SNPs that did not show a significant (*P* < 0.05) association with cystatin C-based eGFR values. Nine SNPs, including smoking status and educational level, were excluded because of significant associations (*P* < 1 × 10^–5^) with an increased risk of COVID-19. Finally, 395 SNPs with a genome-wide significant association with log-transformed eGFRcrea values were used as an exposure (Supplementary Tables 2 and Table 4).

In this study, we performed GWAS meta-analysis of log-transformed eGFR values based on serum levels of cystatin C from individuals of European ancestry (N = 460,826) [21]. Trimming was performed, as described above, to ensure that we only included SNPs that were consistently associated with biomarkers related to kidney function. Consequently, 344 SNPs were validated to be significantly (*P* < 0.05) associated with eGFR, when calculated using the serum levels of creatinine and blood urea nitrogen (BUN), with a consistent direction and were not associated with confounding. Next, we performed secondary analysis using information from these 344 SNPs and the statistics relating to their association with eGFRcys as genetic instruments (Supplementary Table 4). We also used other markers related to kidney function, including urinary albumin-to-creatinine ratio (uACR), microalbuminuria, BUN, and uric acid, from GWAS summary data (http://www.nealelab.is/uk-biobank) [22].

### Bidirectional MR based on summary-level data

Bidirectional MR was first performed using summary-level data from individuals of European ancestry; this allowed us to identify causal estimates between COVID and eGFR. In the summary-level MR, we disregarded SNPs that did not overlap between summary statistics or were palindromic with intermediate allele frequencies. The main method applied in the MR was the fixed-effects inverse-variance weighted (IVW) method. Sensitivity analyses were performed with established MR sensitivity analysis methods. First, MR-Egger regression, which yields pleiotropy-robust causal estimates, was performed with bootstrapped standard errors. Second, we applied the panelized weighted median mode method, which yields valid causal estimates even in conditions when invalid instruments are present. Finally, MR-PRESSO was performed; this method detects and corrects the effects of outliers, yielding causal estimates that are robust to heterogeneity. However, the weighted median method is known to be sensitive to the addition or removal of genetic variants. Furthermore, the MR-Egger test is less efficient than the IVW method while the MR-PRESSO has a high false-positive rate with several invalid instrumental variables. We assess the biological functions in the gene set enrichment analysis of eGFR and COVID-19 obtained from Gene Ontology (GO) gene sets. The MR analysis was performed using the TwoSampleMR package in the R environment (version 4.3.1, the R foundation).

## Results

### Causal estimates of the effect of reduced eGFR, as predicted by genetic analysis, on COVID‑19

**Fig. 2:**
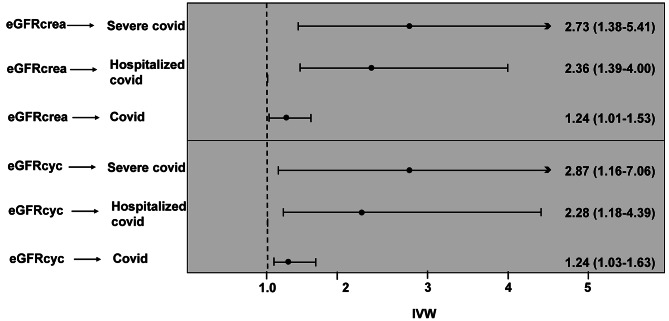
IVW estimates for per 1 unit of log transformed eGFR lower on COVID-19

Initially, 395 SNPs were identified to be associated with eGFRcrea. After excluding SNPs that were likely to be related to the metabolism of creatinine rather than kidney function, and the risk factors for COVID-19 identified by the GWAS meta-analysis of a European population, three SNPs did not overlap with the summary statistics, two SNPs were significantly associated with exposure (*P* < 5E − 5), and nine SNPs were removed because they were palindromic with intermediate allele frequencies. The reduced log-eGFRcrea, as predicted by genetic analysis and the 381 SNPs was significantly associated with a higher risk of severe COVID-19 (odds ratio [OR]-IVW = 2.73; 95% confidence interval [CI], 1.38–5.41; *P* = 0.004) (Fig. 2; Table 1). Furthermore, causal estimates remained significant for the MR-PRESSO test and the with regards to the panelized weighted median (*P* < 0.05). Without reducing the number of SNPs in the genetic instrument (Table 1), the genetic predisposition for reduced log-eGFRcrea remained significantly associated with a higher risk of severe COVID-19. Notably, the genetically predicted reduced log-eGFRcrea was significantly associated with a higher risk of hospitalized COVID (OR-IVW = 2.36; 95% CI, 1.39–4.00, *P*< 0.05) and all COVID cases (OR-IVW = 1.24; 95% CI, 1.01–1.53, *P*< 0.05) (Fig. 2). Other implementations of MR methods are presented in the supplementary material. The genetically predicted reduced log-eGFRcys exhibited the same relationship with COVID-19 (Fig. 2). Causal estimates between eGFRcrea and different phenotypes of COVID-19 infection are shown in Supplementary Table 5. The top 10 significant GO pathways (biological process, cellular component and molecular function) are shown in Supplementary Fig. 2.

**Table 1:** Causal estimates from the summary-level data-based MR from the GWAS summary statistics

Geneticallypredicted exposure	Outcome	MR method	Withtrimmedgenetic instrument			With total SNPs		
			beta	Standard error	*P*	beta	Standard error	*P*
eGFRcrea	Severe COVID-19	Inverse variance weighted	1.01	0.349	0.004	0.831	0.296	0.005
		MR-Egger	1.28	0.806	0.114	1.09	0.66	0.101
		Panelized weighted median	1.38	0.557	0.013	0.615	0.491	0.21
		MR-PRESSO	1.01	0.349	0.004	0.831	0.296	0.005
Severe COVID-19	eGFRcrea	Inverse variance weighted	-0.0002	0.001	0.733	-0.0001	0.0006	0.963
		MR-Egger	-0.0003	0.001	0.814	-0.0002	0.001	0.887
		Panelized weighted median	-0.0001	0.001	0.858	0.0003	0.001	0.626
		MR-PRESSO	-0.0002	0.001	0.736	-0.0001	0.0006	0.963
Severe COVID-19	CKD	Inverse variance weighted	0.015	0.015	0.318	0.019	0.014	0.188
		MR-Egger	0.02	0.027	0.458	0.020	0.027	0.468
		Panelized Weighted median	-0.01	0.02	0.633	0.010	0.020	0.620
		MR-PRESSO	0.015	0.015	0.325	0.019	0.014	0.196

MR = Mendelian randomization, SNP = single nucleotide polymorphism, eGFR = estimated glomerular filtration rate, CKD = chronic kidney disease

### Causal estimates of the effect of genetically predicted COVID‑19 on eGFR and CKD

**Fig. 3:**
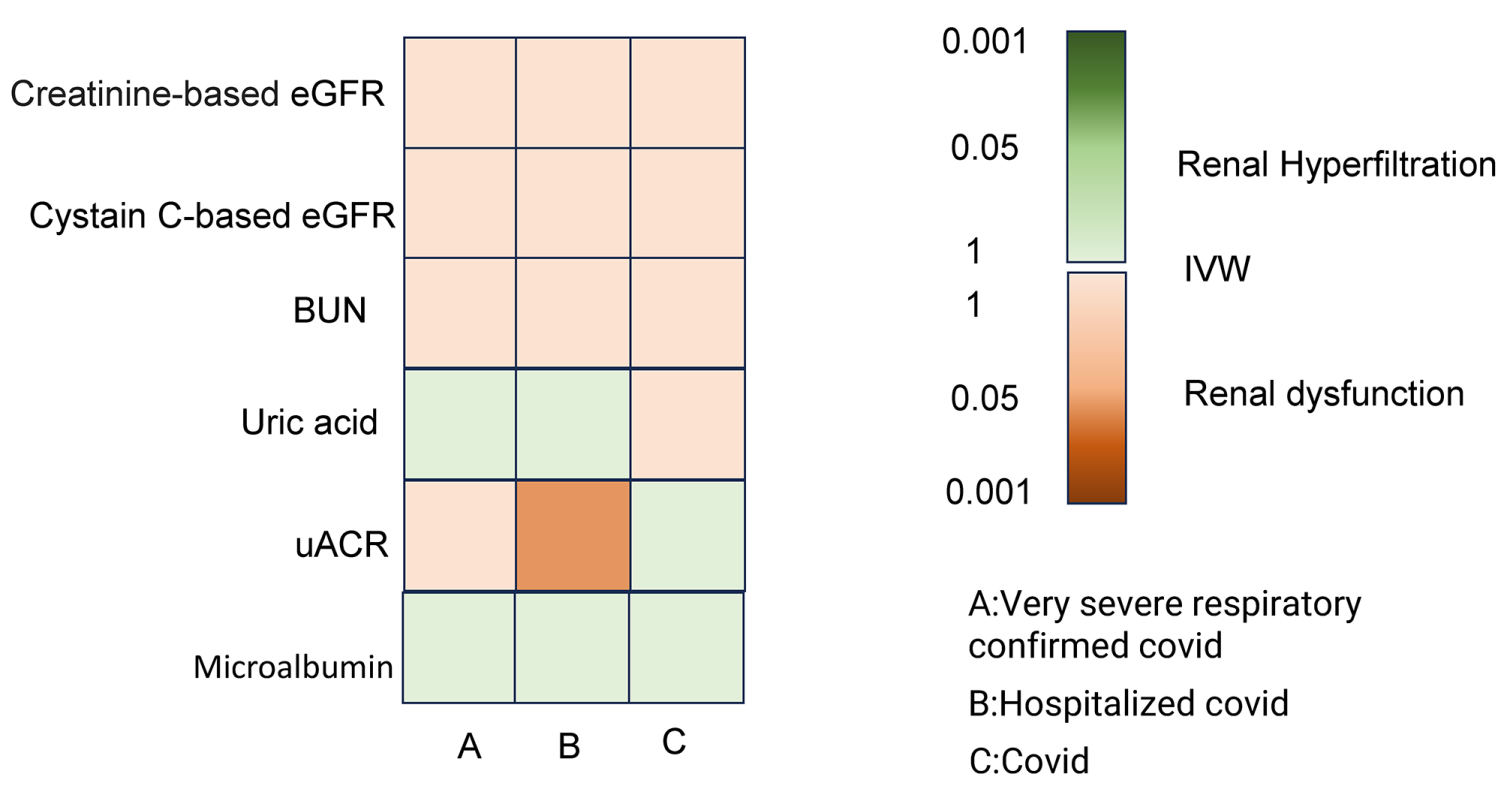
IVW estimates of the effect of COVID-19 on kidney function

MR analysis did not reveal any association between genetically predicted COVID-19 and eGFRcrea (all COVID-19 cases vs. population controls; OR-IVW = 0.997; 95% CI, 0.993–1.002; *P* = 0.211), COVID-19 hospitalization (COVID-19 hospitalization vs. population controls; OR-IVW = 0.999; 95% CI, 0.996–1.001; *P* = 0.193), or severe COVID-19 (severe COVID-19 vs. population controls; OR-IVW = 1.000; 95% CI, 0.999–1.001; *P* = 0.733) (Supplementary Table 6). Effect estimates for the association between COVID-19 and kidney function indicators, including uACR, microalbuminuria, BUN, cystatin C, and uric acid, are shown in Fig. 3. Only hospitalized COVID-19 was associated with a causal increase in the level of uACR (OR-IVW = 1.07, *P* = 0.01). We did not detect a causal relationship between different phenotypes of COVID-19 infection and eGFRcrea/CKD, as shown in Table 2.

**Table 2:** Causal estimates of genetically predicted COVID‑19 on eGFR and CKD from the summary-level data-based MR from the GWAS summary statistics

MR method	Genetically predicted exposure	Outcome	beta	Standard error	*P*	Outcome	beta	Standard error	*P*
Inverse variance weighted	Critical COVID-19 vs. general population	eGFRcrea	-0.0002	9.39E-05	0.109	CKD	-0.001	0.001	0.189
MR-Egger			-0.000225	0.0003	0.463		-0.001	0.004	0.770
Panelized weighted median			-0.0001	4.37E-05	0.999		0.000	0.001	0.950
MR-PRESSO			-0.0001	3.39E-05	0.159		-0.001	0.001	0.434
Inverse variance weighted	Severe COVID-19 with respiratory failure vs. general population	eGFRcrea	-0.0003	0.0003	0.357	CKD	0.008	0.010	0.393
MR-Egger			-0.0001	0.0008	0.896		0.014	0.023	0.567
Panelized weighted median			-0.0002	0.0003	0.538		-0.006	0.011	0.615
MR-PRESSO			-7.87E-05	0.0003	0.765		0.008	0.010	0.406
Inverse variance weighted	Severe COVID-19 vs. general population	eGFRcrea	-0.0005	0.0004	0.244	CKD	-0.008	0.013	0.526
MR-Egger			-0.0008	0.001	0.490		-0.044	0.034	0.200
Panelized Weighted median			-0.0001	0.0005	0.999		-0.010	0.016	0.516
MR-PRESSO			-0.0005	0.0003	0.172		-0.008	0.013	0.530
Inverse variance weighted	Hospitalized COVID-19 vs. non-hospitalized COVID-19	eGFRcrea	0.0002	0.0009	0.684	CKD	0.013	0.010	0.201
MR-Egger			0.0016	0.0011	0.145		0.046	0.019	0.023
Panelized Weighted median			0.00001	0.0004	0.999		0.006	0.014	0.688
MR-PRESSO			0.00018	0.000253	0.484		0.013	0.010	0.211
Inverse variance weighted	Hospitalized COVID-19 vs. general population	eGFRcrea	0.0001	0.0004	0.786	CKD	0.024	0.012	0.055
MR-Egger			-0.0002	0.0008	0.841		0.025	0.027	0.363
Panelized Weighted median			-7.91E-05	0.0005	0.876		0.022	0.017	0.196
MR-PRESSO			-9.80E-05	0.0003	0.773		0.024	0.012	0.060
Inverse variance weighted	COVID-19 vs. general population	eGFRcrea	-0.0010	0.001	0.327	CKD	0.014	0.025	0.560
MR-Egger			-0.0004	0.002	0.863		-0.006	0.056	0.916
Panelized Weighted median			-0.0001	0.001	0.999		-0.015	0.031	0.635
MR-PRESSO			0.0007	0.001	0.338		0.014	0.025	0.564

## Discussion

In the present study, MR analysis demonstrated that genetically instrumented kidney dysfunction (based on eGFRcrea and eGFRcys) was related to a higher risk of severe COVID-19, COVID-19 hospitalization, and all COVID-19 cases. Our findings further support the fact that improving kidney function is likely to exert a beneficial effect on lowering the risk of COVID-19, regardless of phenotype, with significant implications for healthcare and drug repositioning. Furthermore, we revealed that COVID-19 has a complex effect on various indicators of renal function indicators, meaning that the influence of COVID-19 on renal function might be complex.

The kidneys perform a range of functions and are associated with immune responses, inflammation, coagulation, and endothelial function [23–25]. The failure of renal function may result in impaired protein catabolism and the accumulation of metabolic waste products; these effects may contribute to increased inflammation and immunosuppression [26]. Chronic kidney disease may contribute to the infection and development of COVID-19 *via* both direct and indirect mechanisms. Known predisposing factors include immune response dysfunction in the pre-uremic or uremic state and the loss of antibody and complement caused by glomerular damage, systemic inflammation, and immunosuppressants [27]. In a previous study, Mehta et al. reported that the most critical feature of severe COVID-19 is cytokine storm syndrome and immunosuppression. In addition, hypercoagulation and venous thrombosis usually coexist with kidney failure [24, 25, 28]. A previous study reported thrombin production in patients on dialysis and that this effect increased the risk of thrombosis and COVID-19 [29]. Another study found that reduced renal function was associated with endothelial dysfunction [23] and that this effect may also contribute to the development of severe COVID-19. However, these pathways have not been confirmed by experimental studies and cannot be evaluated by MR studies because relevant GWAS data are unavailable.

In a recent MR study [12], Zhao et al. reported that genetic predisposition to eGFRcrea was associated with increased susceptibility to COVID-19. However, as the serum levels of creatinine are known to be influenced by muscle mass and diet, the eGFR value based on cystatin C level has certain advantages when assessing kidney function [14, 15]. Our current findings are preliminary and need to be interpreted cautiously. The protective association of kidney function with severe COVID-19 might reflect an association that is specific to severe COVID-19, an incidental finding, or a lack of power for other COVID-19 outcomes. A previous GWAS meta-analysis of eGFRcrea data that was conducted by the CKDGen Consortium explained almost 20% of the genetic heritability of eGFRcrea [30]. A substantial fraction of missing heritability is expected to be attributed to low-frequency and rare variants, which require even larger GWAS sample sizes. While eGFRcrea is a useful biomarker for kidney function in clinical practice, serum creatinine is a key metabolite of muscle metabolism and, thus, might reflect functions that are not specific to the kidney. It is very difficult for eGFRcrea GWAS to identify mechanisms of biomarker metabolism from modulators of kidney function. The estimation of GFR by serum cystatin C may represent a better marker of GFR. However, eGFRcys can also be influenced by factors other than GFR, such as inflammation, obesity and diabetes. Moreover, eGFRcys has a limited role in the GWAS of kidney function due to high costs and small datasets [14, 15, 31]. Therefore, in the present study it was necessary to perform additional trimming of SNPs to remove genetic variants that were likely to be related to the metabolism of creatinine instead of kidney function.

In the present study, we conducted updated bidirectional MR analysis, including genome-wide summary statistics for log-eGFRcrea or eGFRcys with a large sample size and utilizing the very latest GWAS data relating to COVID-19. In the present study, we found that genetically instrumented better kidney function (based on lower eGFRcrea and eGFRcys values) was significantly related to a higher risk of severe COVID-19, COVID-19 hospitalization, and all COVID-19 cases. We defined severe COVID-19 as death and respiratory support following hospitalization due to COVID-19, in which hospitalization involved supplemental oxygen (not including simple supplementary oxygen), non-invasive mechanical ventilation, and invasive mechanical ventilation. The genetic associations with severe COVID-19 were derived from summary statistics; it was not possible to provide a breakdown by the mode of respiratory support. In addition, we investigated the impact of eGFR on severe COVID-19 with respiratory failure and critical COVID-19. However, due to the limited number of cases in the fourth round and the absence of phenotypic data in the seventh round of GWAS summary data for COVID-19, we were unable to acquire meaningful results. However, we found that a reduced eGFR was causally associated with an increase in the risk of COVID-19 infection and hospitalization. Therefore, our findings indicate that COVID-19 and kidney function are closely linked; this is a major problem since COVID-19 remains a persistent threat to public health across the world. The ongoing COVID-19 pandemic demands a concerted effort to develop new therapeutic strategies to prevent new infections and reduce the severity of infection in patients.

In this study, we investigated the association between COVID-19 and multiple indices of kidney function. However, we obtained inconsistent conclusions, potentially due to the influence of different metabolic pathways on the selected indices or the complex effects of COVID-19 that might lead to renal hyperfiltration or renal dysfunction. Several cross-sectional studies have demonstrated that renal hyperfiltration is associated with various medical conditions, including diabetes, hypertension, obesity, prehypertension, and prediabetes, as well as lifestyle factors, such as smoking, the lack of physical activity, and low levels of aerobic physical activity [32–34]. We identified a causal association between hospitalization for COVID-19 and an increase in uACR; however, these results need to be interpreted with caution. Further analysis of specific individuals is now necessary to fully determine the effect of COVID-19 on renal function. A previous observation study reported that kidney dysfunction is common in patients with COVID-19 and may result in a progressive decline in kidney function and the onset of CKD [35]. Thus, patients with COVID-19 and kidney impairment should be managed in an active manner to protect kidney function and minimize potential long-term effects.

### Limitations

There were several limitations to this study that need to be considered. First, because the COVID-19 pandemic is ongoing and COVID19-hg GWAS meta-analyses round 7 including UK Biobank, it is inevitable that our analysis may have featured a certain overlap in samples that may have caused bias in the two-sample MR. However, our analysis included many SNP sites and our MR analysis was performed in a rigorous manner. Second, although we demonstrated the causal effect of eGFR on COVID-19, dedicated clinical trials are required to fully determine whether the risk of COVID-19 can be actually reduced *via* the management of kidney dysfunction. Furthermore, it is important that future studies investigate non-genetic influences and other potential confounders that were not accounted for in the present study. Third, it is possible that a non-linear causal relationship may exist between COVID-19 and the changes of eGFR; to address this, it is necessary to perform non-linear MR analysis for individual patients. Finally, our MR analysis only involved a population of patients of European ancestry; consequently, our current findings now need to be verified in populations of patients from other ancestries.

## Conclusion

Our MR analysis demonstrated that kidney function plays a stronger role in COVID-19 than the serum levels of cystatin C or creatinine. Regardless of the severity of COVID-19, kidney function appears to be one of the key targets of COVID-19. Investigation of the underlying pathways and the use of available medications that improve kidney function, such as antihypertensives, might be beneficial for the treatment of COVID-19 treatment, with relevance to drug repositioning and healthcare. Moreover, we found that COVID-19 exerts a complex effect on various renal function indices, necessitating further research to investigate the specific mechanisms and pathways that underlie these effects.

### Electronic supplementary material

Below is the link to the electronic supplementary material.

Supplementary Material 1. Supplementary Table  1 Index SNPs significantly associated with potential confounders of COVID-19 in summary-level data-based MR when Genetically predicted eGFR-cr used as exposure. Supplementary Table  2 Genetic instruments for log-transformed eGFR based on creatinine levels. Supplementary Table 3 Index SNPs significantly associated with potential confounders of eGFR-cr in summary-level data-based MR when Genetically predicted COVID-19 used as exposure. Supplementary Table 4. Genetic instruments for log-transformed eGFR based on cystatin C levels. Supplementary Table 5. Causal estimates between eGFRcrea and different COVID-19 infection phenotype from the summary-level data-based MR from the GWAS summary statistics. Supplementary Table 6. Causal estimates between COVID-19 and eGFRcrea from the summary-level data-based MR from the GWAS summary statistics

Supplementary Material 2. Supplementary Fig. 1 Sensitivity analysis of causal association between eGFR and severe COVID-19. Supplementary Fig. 2 Sensitivity analysis of causal association between eGFR and COVID-19 hospitalization. Supplementary Fig. 3 Sensitivity analysis of causal association between eGFR and COVID-19. Supplementary Fig. 4 Top 10 significant GO pathways of eGFR and COVID-19.

## Data Availability

The datasets generated and/or analyzed during the current study are available in the GWAS summary statistics (https://gwas.mrcieu.ac.uk/) and COVID-19 Host Genetics Initiative (https://www.covid19hg.org/).
