# Supplementary material for: The causal relationship between COVID-19 and estimated glomerular filtration rate: a bidirectional Mendelian randomization study
Source: BMC Nephrol. 2024 Jan 15;25:21. doi: 10.1186/s12882-023-03443-4 (PMC10790484; doi:10.1186/s12882-023-03443-4)
Supplement: Supplementary file 2 — Supplementary Material 2. Supplementary Fig. 1 Sensitivity analysis of causal association between eGFR and severe COVID-19. Supplementary Fig. 2 Sensitivity analysis of causal association between eGFR and COVID-19 hospitalization. Supplementary Fig. 3 Sensitivity analysis of causal association between eGFR and COVID-19. Supplementary Fig. 4 Top 10 significant GO pathways of eGFR and COVID-19. [file 12882_2023_3443_MOESM2_ESM.docx]

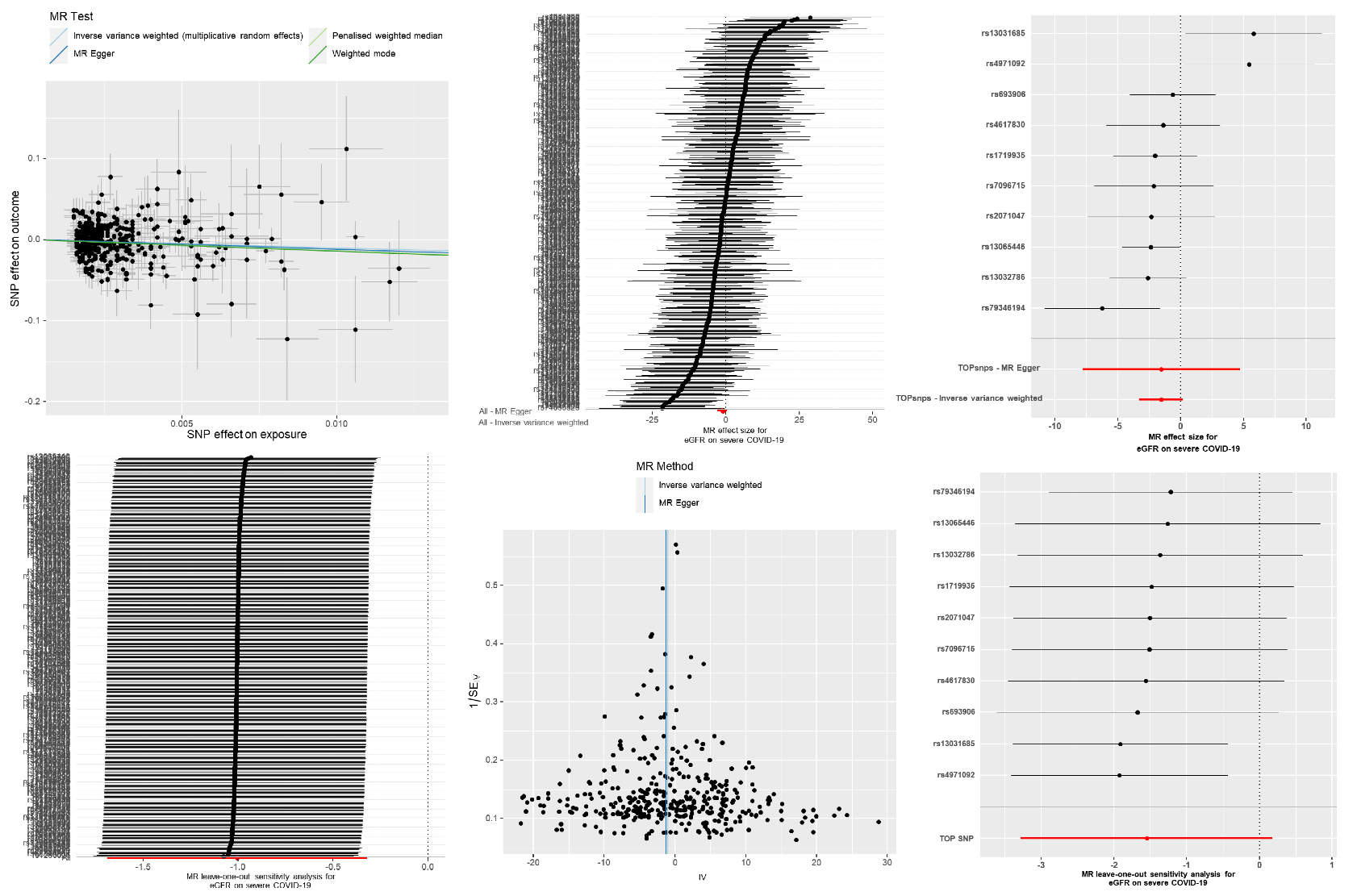


Supplement Figure 1 Sensitivity analysis of causal association between eGFR and severe COVID-19


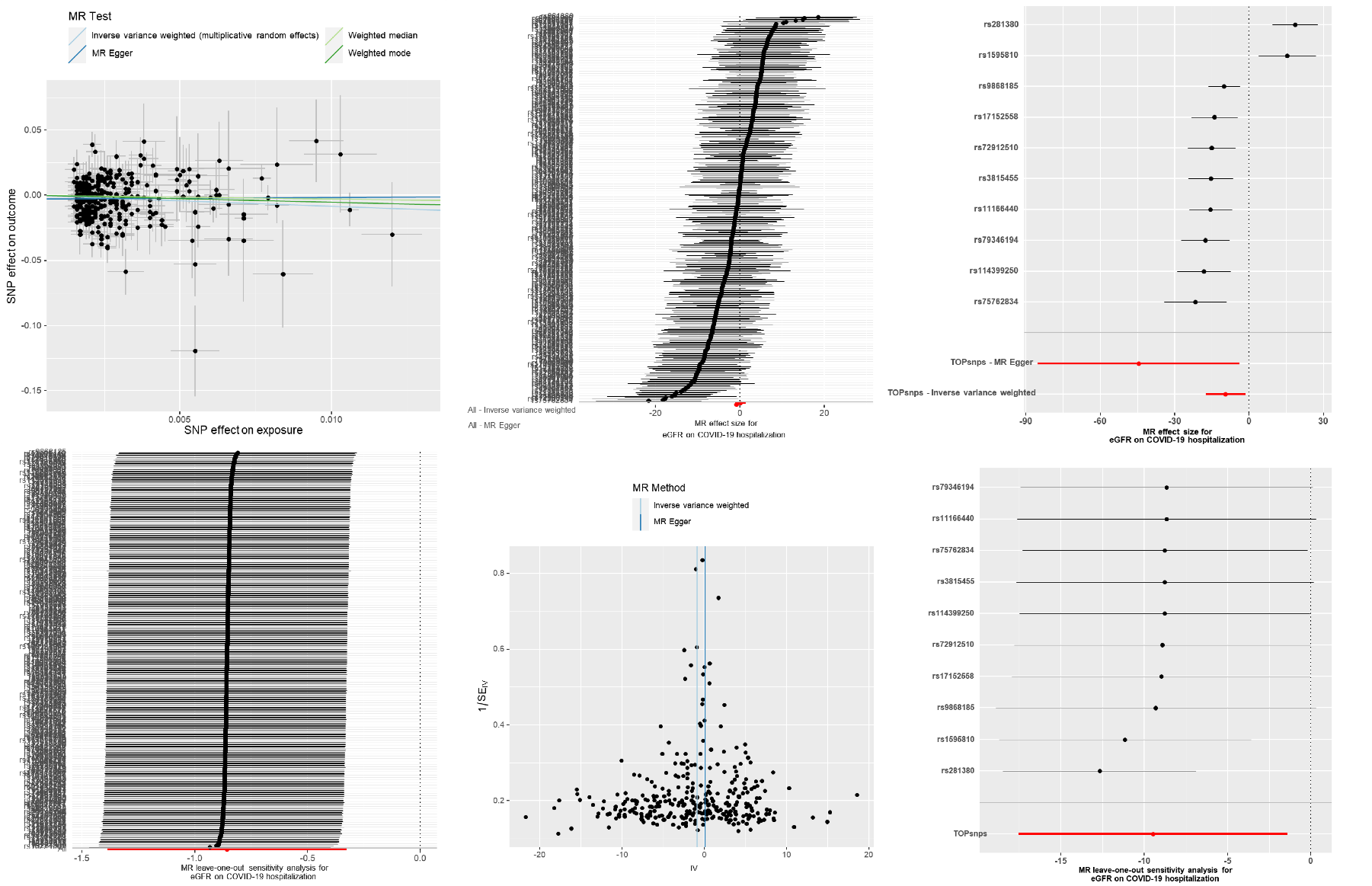


Supplement Figure 2 Sensitivity analysis of causal association between eGFR and COVID-19 hospitalization


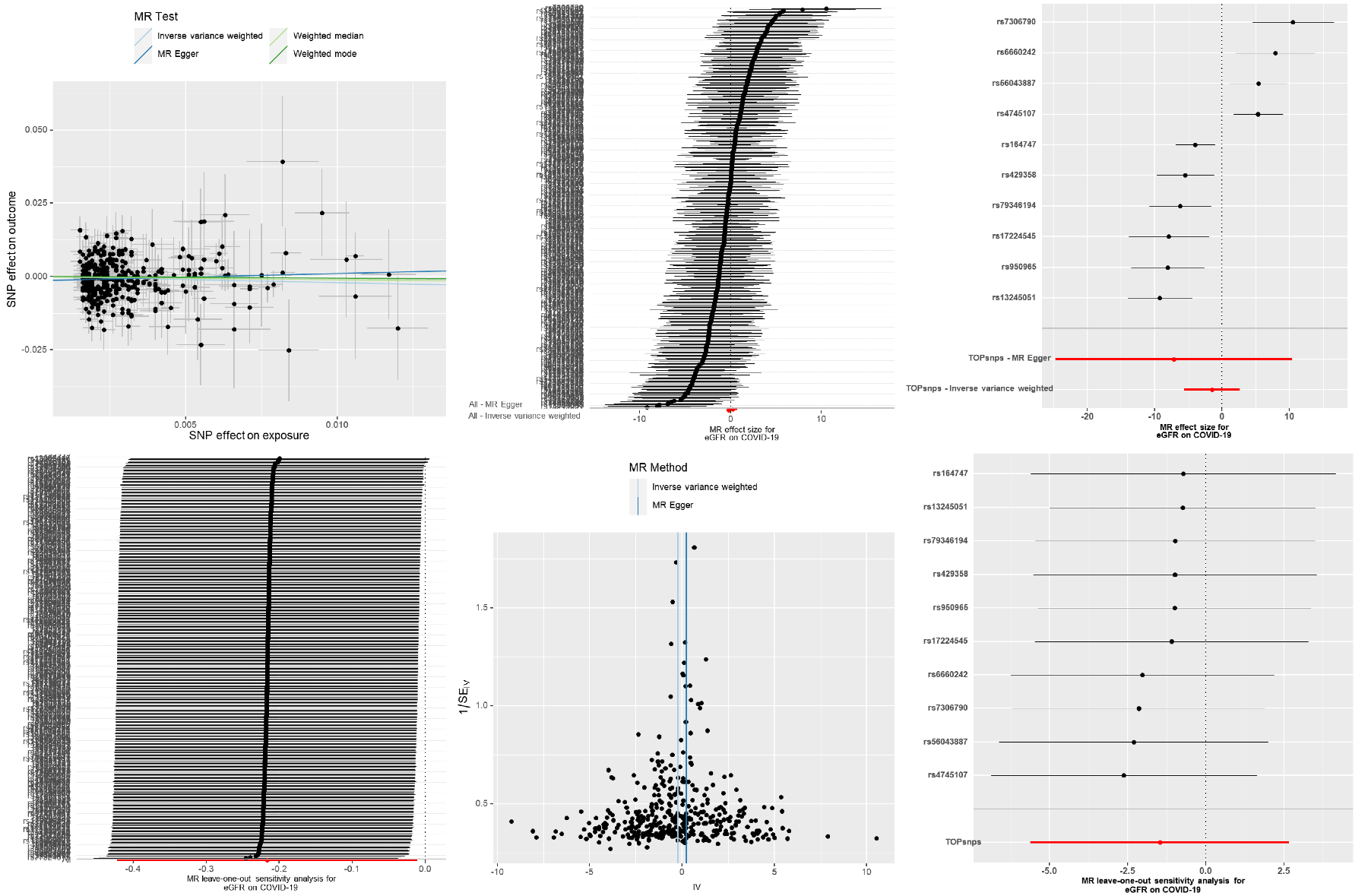


Supplement Figure 3 Sensitivity analysis of causal association between eGFR and COVID-19


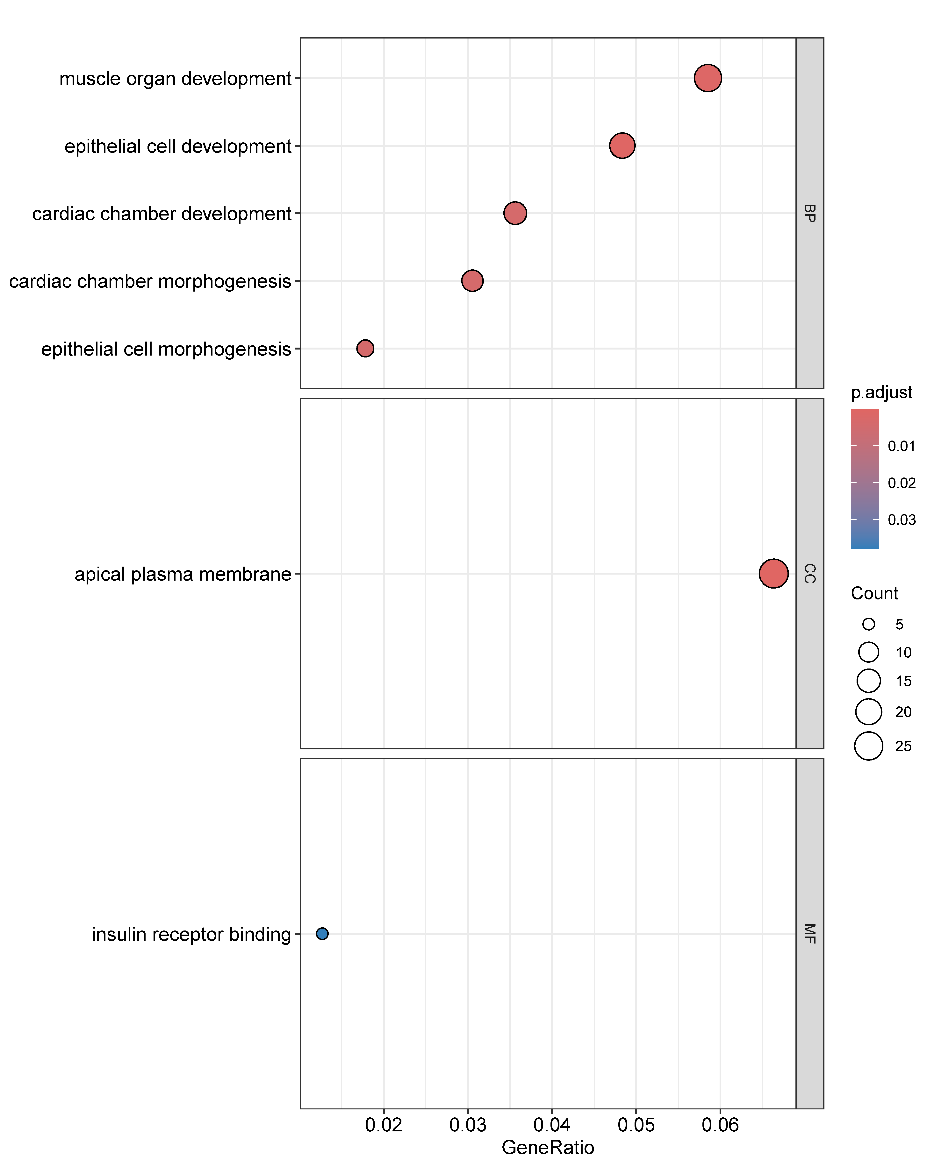


Supplement Figure 4 Top 10 significant GO pathways of eGFR and COVID-19
